# Combination therapy targeting integrins reduces glioblastoma tumor growth through antiangiogenic and direct antitumor activity and leads to activation of the pro-proliferative prolactin pathway

**DOI:** 10.1186/1476-4598-12-144

**Published:** 2013-11-20

**Authors:** Leticia Oliveira-Ferrer, Jasmin Wellbrock, Udo Bartsch, Eva Maria Murga Penas, Jessica Hauschild, Marianne Klokow, Carsten Bokemeyer, Walter Fiedler, Gunter Schuch

**Affiliations:** 1 Department of Oncology, Hematology and Bone Marrow Transplantation with Section Pneumology, University Cancer Center Hamburg (UCCH), Universtity Medical Center Hamburg-Eppendorf, Hamburg, Germany; 2 Department of Gynecology University Cancer Center Hamburg (UCCH), University Medical Center Hamburg-Eppendorf, Hamburg, Germany; 3 Department of Ophthalmology, University Medical Center Hamburg-Eppendorf, Hamburg, Germany; 4 Institute of Human Genetics, Christian-Albrechts-University of Kiel & University Hospital Schleswig-Holstein, Campus Kiel, Kiel, Germany

**Keywords:** Gliobastoma, Angiogenesis, Endostatin, Tumstatin, Prolactin receptor

## Abstract

**Background:**

Tumors may develop resistance to specific angiogenic inhibitors via activation of alternative pathways. Therefore, multiple angiogenic pathways should be targeted to achieve significant angiogenic blockade. In this study we investigated the effects of a combined application of the angiogenic inhibitors endostatin and tumstatin in a model of human glioblastoma multiforme.

**Results:**

Inhibitors released by stably transfected porcine aortic endothelial cells (PAE) showed anti-angiogenic activity in proliferation and wound-healing assays with endothelial cells (EC). Interestingly, combination of endostatin and tumstatin (ES + Tum) also reduced proliferation of glioma cells and additionally induced morphological changes and apoptosis *in vitro*. Microencapsulated PAE-cells producing these inhibitors were applied for local therapy in a subcutaneous glioblastoma model. When endostatin or tumstatin were applied separately, *in vivo* tumor growth was inhibited by 58% and 50%, respectively. Combined application of ES + Tum, in comparison, resulted in a significantly more pronounced inhibition of tumor growth (83%). cDNA microarrays of tumors treated with ES + Tum revealed an up-regulation of prolactin receptor (PRLR). ES + Tum-induced up-regulation of PRLR in glioma cells was also found in *in vitro*. Moreover, exogenous PRLR overexpression *in vitro* led to up-regulation of its ligand prolactin and increased proliferation suggesting a functional autocrine growth loop in these cells.

**Conclusion:**

Our data indicate that integrin-targeting factors endostatin and tumstatin act additively by inhibiting glioblastoma growth via reduction of vessel density but also directly by affecting proliferation and viability of tumor cells. Treatment with the ES + Tum-combination activates the PRLR pro-proliferative pathway in glioblastoma. Future work will show whether the prolactin signaling pathway represents an additional target to improve therapeutic strategies in this entity.

## Background

Human glioblastoma multiforme (GBM) is the most common and malignant type of brain tumors. Current treatment options such as surgical intervention, radiation therapy or cytotoxic chemotherapy do not significantly improve the median survival beyond approximately 12 to 18 months for patients with GBM [1,2]. Therefore, the identification and the development of novel and more efficient therapeutic approaches remain a crucial task for this disease.

Since GBM is characterized by particularly high levels of neovascularization, a therapeutic strategy based on angiogenic blockade appears to be promising. Actually, a number of strategies targeting new blood vessel formation have shown some success in preclinical models of GBM [3,4] and several clinical trials with anti-angiogenic agents are ongoing [5]. An important feature of angiogenesis is the interaction of endothelial cells (EC) with surrounding extracellular matrix (ECM). Integrin binding mediates cell adhesion of ECs to surrounding ECM and regulates their survival, growth and mobility [6]. Integrins and αVβ5 are predominantly expressed in proangiogenic ECs [7,8] and especially integrin αVβ3 has been found to be upregulated in ECs of GBM tumors [9,10]. Cilengitide, a cyclic pentapeptide mimicking the Arg-Gly-Asp (RGD) binding site of integrin ligands, was identified as a potent and selective integrin antagonist that interfered with binding of ECM components to αVβ3 and αVβ5 integrins [11]. In preclinical models cilengitide had synergistic therapeutic effects with radioimmunotherapy in breast cancer and orthotopic brain tumor models [12,13]. However, expression of αVβ3 and αVβ5 integrins is not restricted to activated ECs. Both integrins are also in brain tumor cells [14-16]. In fact, we have recently shown that cilengitide inhibits integrin-dependent signaling and induces apoptosis not only in endothelial but also in glioma cells thereby explaining the profound activity of integrin inhibitors in this disease [17]. These data suggest that anti-angiogenic molecules directed towards integrins may have a multi-targeting effect on both endothelial and glioma cells.

An additional aspect to be considered for the design of novel therapeutic strategies against GBM is the ability of these tumors to escape anti-angiogenic monotherapy [18,19]. Therefore, it might be necessary to target multiple pro-angiogenic pathways in order to achieve significant anti-tumorigenic effects.

Here, we studied two angiogenic inhibitors targeting different angiogenic pathways, endostatin (ES) and tumstatin (Tum), and evaluated the anti-tumorigenic activity of the individual factors and a combination of both factors in an in vivo model of GBM. ES has been reported to interfere with integrin α5β1 and VEGFR-2 in ECs, while Tum binds αvβ3 and αVβ5 integrins and induces apoptosis in ECs [20,21]. In addition, microarray analysis of tumor tissue was performed to identify activation of alternative pro-tumorigenic signalling pathways in tumor cells.

## Results

### Encapsulation of stably transfected PAE cells expressing angiogenic inhibitors and functional analyses in vitro

**Figure 1:**
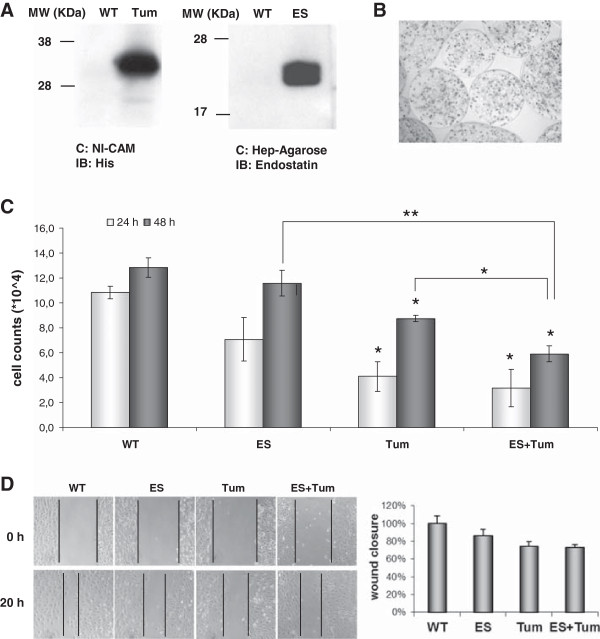
**Effects of conditioned medium from encapsulated PAE cells overexpressing ES and Tum. (A)** Secretion of ES and Tum from stably transfected PAE cells was determined using Western blot analysis of culture supernatants after protein concentration with heparin sepharose for ES and Nickel Cam for Tum. **(B)** Transfected PAE cells were encapsulated in alginate/PLL as described. Phase contrast photomicrograph shows transfected cells in microbeads at a magnification of x10. **(C)** HUVECs cultivated in CM containing ES, Tum or ES + Tum showed reduced proliferation rates when compared with cells cultivated in CM from PAE WT cells. Bars represent mean values ± SE (*n* = 3); * on bars indicates significant differences vs. WT (p < 0.05); */** on connecting lines indicates significant differences between respective groups (p < 0.05 / p < 0.005). **(D)***Wound healing assay.* CM containing ES, Tum or ES + Tum reduced migration of HDMECs. Wound closure data are normalized to results obtained with CM from PAE WT cells.

The expression of ES and Tum in the CM from stably transfected PAE cells was confirmed by Western blot analysis (Figure 1A). After cell encapsulation [22,23], cells in the alginate microbeads were cultured for several weeks, and the CM analysed by Western blot after different culture periods to confirm continuous release of angiogenic inhibitors (Figure 1B).

Next, we tested the functionality of the inhibitors secreted by the stably transfected PAE cells in proliferation and wound assays on endothelial cells. CM from transfected cells decreased proliferation of HUVECs *in vitro* when compared to CM from WT cells (Figure 1C). We observed a moderate reduction on cell proliferation of ECs incubated with ES containing medium. In comparison, CM from Tum transfected cells strongly reduced EC numbers to approximately 60% and 35% after 24 and 48 hours, respectively. Next, CM from PAE-WT, -ES, and -Tum cells were used in a wound assay *in vitro*. Compared to CM from WT control cells, media containing the inhibitors decreased wound closure to 13%, 25% and 27% for ES, Tum, and ES + Tum, respectively (Figure 1D).

### Effect of angiogenic inhibitors on glioma cells

In order to analyse whether angiogenic inhibitors exert direct effects on glioma cells we performed *in vitro* cell proliferation and apoptosis assays. Glioma cells and particularly the periphery of high-grade gliomas are known to express integrins [9]. In line with these data, expression analyses at the mRNA and protein level of the human glioma cell line G55 showed expression of αVβ3 and α5β1 integrins. (Additional file 1: Figure S1; supplementary data).

**Figure 2:**
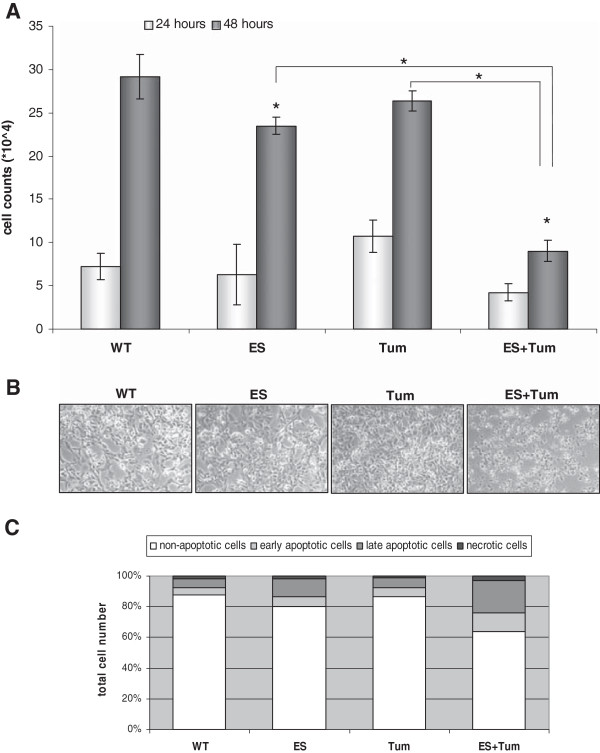
**Conditioned medium containing ES + Tum reduced proliferation and induced apoptosis in G55 glioma cells *in vitro*. (A)** G55 cells were cultivated in CM from PAE WT cells, and in CM containing ES or Tum or ES + Tum. Cell numbers were determined after a culture period of 24 and 48 hours. Bars represent the means ± SE (*n* = 3); * on bars indicates significant differences vs. WT; * on connecting lines indicates significant differences between respective groups (p < 0.05). **(B)** Phase contrast micrographs of G55 cells after a culture period of 24 hours in CM from PAE-WT cells or CM containing ES or Tum or ES + Tum. **(C)** Quantification of apoptotic G55 cells after cultivation in CM from PAE WT cells or CM containing ES or Tum or ES + Tum using flow cytometric analysis after FITC-conjugated annexin-V and PI staining.

Treatment of G55 cells with CM containing either ES or Tum had only weak inhibitory effects on cell proliferation. In contrast, CM containing ES + Tum remarkably reduced G55 cell proliferation to 60-65% compared to CM containing ES or Tum, alone after 48 hours (Figure 2A). To evaluate cell viability in response to angiogenic inhibitors, G55 cells were analyse with phase-contrast microscopy and cell apoptosis was measured using Annexin V/Propidium Iodid staining by FACs 24 hours after treatment. As shown in Figure 2B, G55 cells presented a normal morphology when cultured in CM from PAE-WT, PAE-Tum or PAE-ES. In contrast, G55 cells treated with CM containing ES + Tum did not proliferate and displayed striking morphological changes such as flattening and cell detachment. Notably, ES + Tum induced similar morphological changes in the glioma cell lines G44 and G28 (data not shown). CM from ES- or Tum-transfected cells did not induce increased apoptotic death of G55 cells when compared to CM from WT cells. When cultures were treated with CM containing ES + Tum, in contrast, the frequency of apoptotic G55 cells was significantly increased by about 23% when compared to G55 cultures treated with CM from WT control cells (Figure 2C).

### Locally implanted microbeads inhibit subcutaneous tumor growth

**Figure 3:**
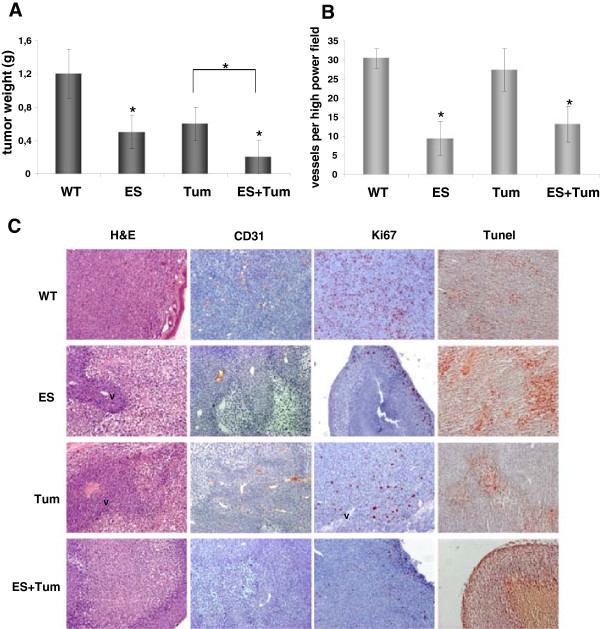
**Analysis of G55 tumors treated with ES**, **Tum and ES** + **Tum. Effect on tumor growth and tumor vessel density. (A)** Subcutaneously grafted tumors were treated with ES, Tum and ES + Tum for 10 days, tumors were excised and tumor weights determined. Bars represent the mean values ± SE (n = 5); * on bars indicates significant differences vs. WT; * on connecting lines indicates significant differences between respective groups (p < 0.05). **(B)** Microvessel density was analysed by counting CD31-positive vessels in tumor tissue. Note that microvessel density is significantly reduced in ES- and ES + Tum-treated tumors, but not in Tum-treated tumors. Bars represent the means ± SE (n = 4-5); * significant differences vs. WT (p < 0.05). **(C)** Tumor tissue was analysed by H&E, TUNEL and immunohistochemical stainings for Ki67 and CD31. ES-, Tum- and ES + Tum-treated tumors showed large necrotic areas containing TUNEL-positive cells. Vital cells were restricted to thin cell layers around vessels (*v*) and at the outer margins of tumors. Control tumors (WT), in comparison, were highly proliferative and lacked large necrotic areas.

To further investigate the effects of antiangiogenic inhibitors on GBM *in vivo*, G55 cells were grown subcutaneously as xenografts in SCID mice. Tumors were subsequently treated with angiogenic inhibitors alone or in combination using microencapsulation technology as described before [24]. We observed a strong reduction of tumor weight by about 60% and 50% for ES and Tum treated animals, respectively, when compared with the control group. Interestingly, the ES + Tum combination showed the strongest reduction of tumor weight of approximately 83% when compared to control tumors (Figure 3A). Furthermore, blinded analyses of microvessel density showed a significant inhibition of vascularization in ES-treated tumors. Vessel density in Tum-treated tumors, however, was not significantly altered (Figure 3B). The combined application of ES and Tum also resulted in a significantly reduced microvessel density in G55 tumors.

Histological analyses showed highly proliferative tumors with absence of large necrotic areas in control animals. Tum- and ES-treated tumors, in comparison, were characterized by large necrotic areas with numerous TUNEL-positive cells were observed. Vital cells were restricted to thin layers surrounding vessels and to the outer margins of tumors (Figure 3C). A similar pattern was seen in tumors treated with ES + Tum.

### cDNA microarrays and target genes of anti-angiogenic treatment in G55 tumors

**Figure 4:**
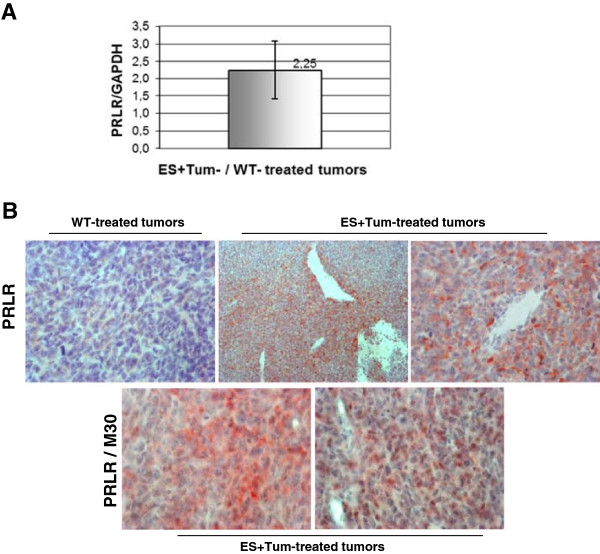
**Expression analyses of prolactin receptor in tumor tissue. (A)** Quantitative RT-PCR revealed a 2.5-fold upregulation of prolactin receptor mRNA expression in ES + Tum-treated tumors when compared to control tumors **(B)***Upper side:* Immunostaining for prolactin receptor in control tumors (x10) and ES + Tum-treated tumors (*left* x10; *right* x40). *Lower side*: Immunostaining for prolactin receptor and cleaved cytokeratin (M30) in ES + Tum-treated tumors (x40).

We next analysed whether treatments with the different angiogenic inhibitors induced responses in glioma cells via differential regulation of alternative cellular pathways. To address this question we performed cDNA arrays with mRNA isolated from tumor tissue after treatment with ES, Tum or ES + Tum. No genes with an at least twofold increase or decrease in expression level (mean signal log ratio ‡1.0) were identified in any of the treated groups. Based on the fact that standard thresholds might be too stringent at high intensities [25], we defined a lower signal log ratio as a threshold and selected some relevant genes for further validation analysis (Additional file 2: Table S1; supplementary data). Of those, we considered prolactin and its receptor appeared as the most interesting candidate genes, since this pathway has been reported to be involved in the progression of several tumor type [26,27]. Prolactin receptor (PRLR) was found to be up-regulated (SLR: +0.3) in tumors treated with the ES + Tum combination when compared to control tumors. The microarray results were verified in identical mRNA samples from tumor tissues analysed by quantitative RT-PCR. Prolactin receptor (PRLR) expression showed a 2.5 fold up-regulation in tumors treated with the ES + Tum combination compared to the control group (Figure 4A). The same tumor material used for microarray analyses was histologically analysed for the expression of PRLR. In contrast to control tumors which displayed low to moderate staining for PRLR, ES + Tum-treated tumors showed intense immunoreactivity for PRLR. PRLR-staining pattern was heterogeneous and mainly localized in certain tumor regions. Double immunohistochemical staining for the apoptosis marker M30 (brown) and PRLR (red) revealed no or few apoptotic cells in areas with high levels of PRLR, whereas M30 positive cells presented low or no PRLR expression (Figure 4B).

### Simultaneous treatment with endostatin and tumstatin of G55 cells in vitro induces PRLR-up-regulation in G55 cells in vitro

**Figure 5:**
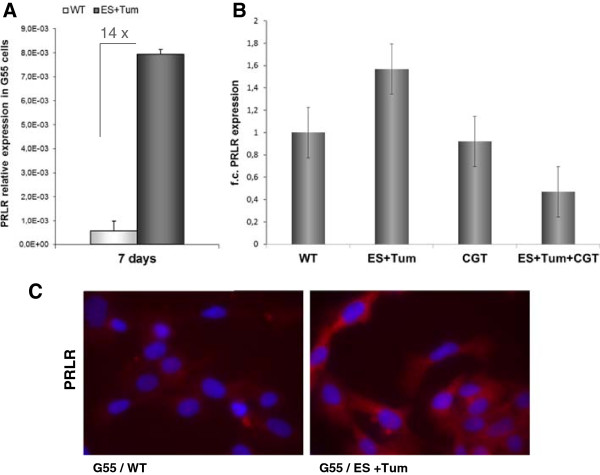
**Elevated levels of PRLR mRNA in glioma cells treated with ES + Tum. (A)** Quantification of prolactin receptor mRNA expression revealed a 14-fold increase in G55 cells treated with CM containing ES + Tum when compared to G55 cells treated with CM from PA-WT cells. **(B)** Quantification of prolactin receptor mRNA expression in G55 cells treated with CM from PAE-WT, CM containing ES + Tum and cilengitide (CGT, 5 μg/ml) alone and in combinations. Bars represent the mean values ± SE from three independent experiments. **(C)** Immunofluorescence staining for PRLR in G55 cells after treatment with CM from PAE-WT and CM containing ES + Tum.

Glioma cells were treated for 7 days with CM from PAE-WT cells or a mixture of CM from ES- and Tum-PAE transfected cells. Subsequent expression analyses at the mRNA level revealed a 14fold up-regulation of PRLR in cells stimulated with ES + Tum when compared with the control cells (Figure 5A). Blockade of integrins αvβ3/αvβ5 with the RGD-peptide cilengitide (CGT; 5 μg/ml) after 3 days did not affect PRLR expression, whereas simultaneous treatment with CGT and the Tum + ES combination blocked the ES + Tum-induced up-regulation of PRLR (Figure 5B). Immunofluorescence analysis on G55 cells showed cell clusters with intensive PRLR staining in those cells treated with ES and Tum, whereas the PRLR level in WT-treated cells remained low (Figure 5C).

### PRLR stimulates proliferation and survival of G55 glioma cells

**Figure 6:**
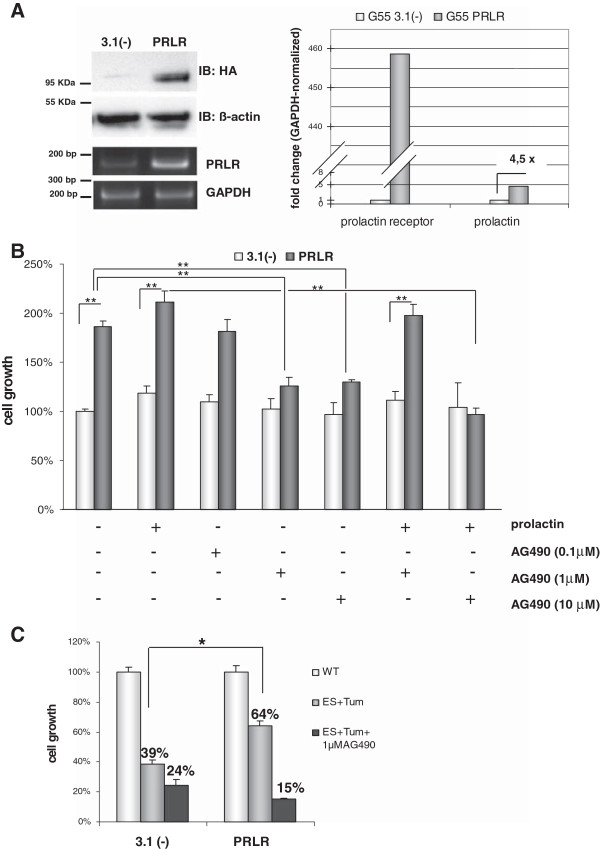
**PRLR overexpression in glioma cells *in vitro*. (A)** Verification of PRLR-overexpression in the glioma cell line G55 using Western blot analysis and RT-PCR (*left*). Quantification of PRLR and PRL mRNA expression in stably PRLR-transfected (G55 PRLR) and mock-transfected ((G55 3.1(−)) G55 cells (*right*). **(B)** Effect of PRLR overexpression on cell growth in the presence or absence of PRL and the Jak-2 inhibitor AG-490. Bars represent the mean values ± SE (n = 5-6); ** on connecting lines indicates significant differences between respective groups (p < 0.005). **(C)** PRLR- and mock- (3.1(−)) transfected G55 cells were treated with CM from PAE-WT or CM containing ES + Tum with and without the Jak-2 inhibitor AG-490. Bars represent the mean values ± SE (n = 5-6); * on connecting lines indicates significant differences between respective groups (p < 0. 05).

To investigate the potential role of PRLR in glioma tumor cells, we examined the expression levels and functionality of endogenous PRLR in two glioma cell lines (G28 and G55). We detected PRLR mRNA expression in both G28 and G55 cells (Additional file 3: Figure S2-A; supplementary data) and observed that prolactin (PRL), the cognate ligand of the PRLR, stimulated cell proliferation of both cell lines in a dose-dependent manner (Additional file 3: Figure S2-B; supplementary data). These data indicates that G28 and G55 cells express a functional PRLR which apparently exerts a pro-proliferative effect. In a second step and mimicking the PRLR-up-regulation in ES + Tum treated tumors *in vivo*, we overexpressed PRLR in G55 cells *in vitro*. Cells were transfected with an expression vector encoding HA-tagged full length PRLR or with the empty vector as a control. Overexpression of PRLR in stably transfected cells was confirmed at the mRNA and protein level as shown in Figure 6A. Interestingly, we observed a 4,5 fold up-regulation in the expression level of prolactin at the mRNA level in cells with forced expression of PRLR (Figure 6A *right*).

The effect of forced expression PRLR in G55 cells growth was further examined using the WST-1 colorimetric assay. Figure 6B illustrates proliferation rates of PRLR-overexpressing versus control cells after 72 hours incubation with prolactin and the inhibitor AG-490 in the absence of serum. Values are given in percent and are related to the control cells that were incubated with basal medium only. Under these conditions PRLR-overexpressing cells showed a significantly increased proliferation activity (86%) when compared to mock-transfected cells. Treatment with the ligand PRL at a concentration of 2 nM induced a minor stimulation of proliferation of control and PRLR-overexpressing cells to an extent of 18% and 25%, respectively. In order to corroborate the PRLR-related increase in cell proliferation, we administered AG-490, a potent inhibitor of the Jak2 tyrosine kinase, which is critical for the transmission of PRLR-mediated proliferative signals [28]. While AG-490 reduced proliferation of PRLR-overexpressing cells in a dose dependent manner, it did not significantly affect proliferation of control cells. Thus, at a concentration of 1 μM AG-490 we observed comparable proliferation rates for PRLR-overexpressing and control cells. In the presence of 2 nM prolactin, higher concentrations of inhibitor AG-490 (10 μM) were necessary to block the PRLR-related increase of proliferation.

Next, we analysed the effect of ES + Tum treatment in cells overexpressing PRLR. To this aim, proliferation rates of PRLR-overexpressing or control cells were determined in CM containing ES + Tum and related to those observed in CM from PAE-WT cells. While ES + Tum dramatically decreased proliferation of control cells to about 39%, proliferation of PRLR-overexpressing cells was reduced to only 64% of the control value (Figure 6C). Additional treatment with the inhibitor AG-490 reduced proliferation rates of both control and PRLR cells to a similar extent (i.e. 24% and 15%, respectively).

## Discussion

Angiogenesis plays a central role in tumor growth and metastasis. Since GBM tumors are highly vascularised, therapeutic strategies based on angiogenic blockade are particularly attractive for this entity. However, it has been observed that initial responses to anti-angiogenic therapy are frequently followed by tumor progression resulting in only limited survival advantage [29-31]. This evasive resistance implies adaptation of tumors to angiogenic inhibitors via activation of alternative pathways that sustain tumor growth.

Accordingly, our approach was designed to simultaneously target different angiogenic signaling pathways and to investigate the activation of possible resistance mechanisms in a GBM model.

Our results show for the first time that the combined application of the integrin inhibitors ES and Tum significantly augment the inhibitory effect over each of the individual substances and that the ES + Tum combination exerts its antitumorigenic effects by both antiangiogenic and direct antitumorigenic activities. Finally, we found an up-regulation of the prolactin receptor in tumor cells treated with the ES + Tum combination and demonstrate a role of this receptor in the control of glioma cell proliferation *in vitro*.

In the present study, the antiangiogenic substances were delivered to a subcutaneous graft of G55 glioma cells using *ex vivo* modified PAE cells, which were encapsulated in alginate microbeads. The microencapsulation technology ensures a continuous release of proteins, and has been successfully used by us and others in different animal models [32,33].

The efficacy of each angiogenic inhibitor was demonstrated on EC proliferation and wound assays *in vitro* and the combination of ES + Tum showed even additive inhibitory effects on endothelial cell proliferation. Local release of single inhibitors ES and Tum by encapsulated PAE cells resulted in inhibition of tumor growth in subcutaneously implanted GBM by about 58% and 50%, respectively, when compared to the control group, respectively. Strikingly, the combined application of ES and Tum inhibited tumor growth by about 83% tumor growth inhibition.

While these observations correlated with a pronounced decrease of vascular density in ES- treated tumors, treatment with Tum resulted in only minimal reduction of blood vessel density, suggesting that *in vivo* tumor growth reduction mediated by Tum is mainly caused by a direct antitumorigenic activities and less through antiangiogenic mechanisms. A direct αVβ3 -dependent growth-inhibitory effect of Tum on glioma cells *in vitro* and *in vivo* has been previously describe by Kawaguchi et al. [34]. On the other hand, the extent of tumor growth inhibition caused by the Es + Tum combination was higher than expected compared with the reduction level of vessel density. This fact prompted us to hypothesize that the ES + Tum combination exerts direct anti-neoplastic effects on glioma cells *in vivo*, in addition to its antiangiogenic effect. This hypothesis was confirmed in our i*n vitro* experiments, which showed reduced proliferation rates of glioma cells after treatment with the ES + Tum combination, but not after treatment with the single inhibitors. Moreover, the ES + Tum combination caused morphological changes and induced apoptosis in glioma cells. Since previous studies have demonstrated that integrin antagonists affect cell cycle progression and viability of glioma cell lines, even inhibiting signaling pathways similar to ECs [34], we suggest that ES and Tum act through their respective integrin receptors on glioma cells, ultimately leading to inhibition of proliferation and induction of apoptosis. Nevertheless, further studies are necessary to clarify the effects of ES + Tum on glioma cells at the molecular level.

In order to gain further insights into possible mechanisms that enable tumor cells to escape anti-angiogenic therapies, we performed cDNA arrays using mRNA from tumor tissue treated with encapsulated PAE-WT cells or PAE cells releasing ES or Tum, either individually or in combination. Surprisingly, we identified only a few genes with a significant increase or decrease in expression level (mean signal log ratio ‡1.0) in the ES-, Tum- or ES + Tum-treated groups when compared with the control group. We focused our interest on the hormone prolactin (PRL) and its cognate receptor PRLR, which were up-regulated after treatment with Tum and ES + Tum, respectively. Validation of PRLR up-regulation in ES + Tum tissue sections by immunohistochemistry revealed a heterogeneous staining pattern with an intensive PRLR staining localized in well-defined tumor regions. Double immunostaining with apoptotic marker M30 and PRLR further showed that those areas with high levels of PRLR contained none or few apoptotic cells, whereas apoptotic regions presented low or no expression of PRLR. Similar results were obtained *in vitro* after immunofluorescence staining for cleaved caspase-3 and PRLR in glioma cells treated with ES + Tum (data not shown). Based on these results, we assume that a subpopulation within the G55 cells does not undergo apoptosis after ES + Tum-treatment but rather proliferates via activation of the PRLR/PRL signaling axis. In Glioblastoma “Cancer Stem Cells” (CSC), a small subpopulation of self-renewing “stem-like” cancer cells, have been demonstrated to show resistance to commonly used anticancer therapies such as radiation [35] and chemotherapy [36,37]. Clark et al. [38] have observed a compensatory activation of multiple ERBB family receptors in GBM CSCs deprived of EGFR signal, suggesting an intrinsic GBM resistance mechanism for EGFR-targeted therapy. To what extent the PRLR-positive subpopulation found in ES + Tum-treated tumor consist of CSC needs to be further investigated in future studies.

Several studies have documented the involvement of the ligand PRL in the growth control of different tumors such as breast [39], liver [40] and prostate [41] and further, PRL antagonists such as hPRL-G129R has been demonstrated to inhibit breast cancer growth *in vitro* and in vivo [42]. However, only little is known about the role of the PRLR/PRL-signaling axis in glioma cells. PRLR expression has been found in rat and human glioma cells [28,43] but also in benign intracranial tumors [44]. Ducret et al. [45] have shown that PRL induces a dose-dependent increase in proliferation and survival of U87-MG glioma cells. In line with these results we have detected PRLR mRNA expression in two additional glioma cell lines (G28 and G55) and could demonstrate that PRL stimulates cell proliferation in a dose-dependent manner, indicating that these cells express a functional PRLR.

Interestingly, we observed a strong up-regulation of PRLR in glioma cells treated with ES + Tum *in vitro*. PRLR expression in contrast was not influenced by oxygen deprivation as observed after incubation of G55 cells under hypoxic and normoxic conditions for 24 hours, 48 hours and 5 days (data not shown). These observations suggest that up-regulation of PRLR in GBM tumors after ES + Tum treatment was not a secondary response to the anti-angiogenic treatment, but rather mediated through direct action of both integrin targeting factors on tumor cells. Although little is known about the effects of ES and Tum on glioma cells at the molecular level, an integrin-mediated auto-regulation of cell proliferation and apoptosis in glioma cells have been recently described by our group and others [17,34]. In addition, an integrin-PRLR cross-talk has recently been described in breast cancer cells [46]. The fact that cilengitide, an integrin αvβ3/αvβ5 inhibitor, partially blocked ES + Tum-mediated effect on PRLR expression point to an integrin dependent mechanism. It is therefore tempting to speculate that the combined application of ES and Tum triggers up-regulation of PRLR in glioma, resulting in augmented PRL signalling and ultimately in increased tumor growth and/or stimulation of angiogenesis [47]. Our *in vitro* data confirm to some extent this hypothesis as they show for the first time that PRLR overexpression significantly increases glioma cell growth. The PRLR-mediated increase of cell growth was abrogated by inhibition of Jak2, a tyrosine kinase that has been described as major downstream regulator of PRLR-signalling [28]. Moreover, we found a 4fold up-regulation of PRL expression in PRLR-overexpressing cells when compared to mock-transfected cells, suggesting a PRL-autocrine loop that stimulates glioma cell growth. Beside the already mentioned pro-proliferative activity of PRLR in diverse tumor entities, several groups have reported about a PRLR/PRL-mediated inhibition of apoptosis especially in response to chemotherapy. In breast cancer cells PRL confers resistance against cisplatin by activating a detoxification enzyme [48,49] and in ovarian carcinoma cells PRL and its receptor inhibit apoptosis induced by serum starvation or cisplatin treatment [49]. These observations might explain the fact that ES + Tum-mediated cell growth inhibition *in vitro* was significantly less pronounced in PRLR overexpressing cells than in control cells.

## Conclusion

Our current data demonstrate that the integrin inhibitors ES and Tum significantly reduce GBM growth *in vivo*. We also demonstrate that a simultaneous application of ES and Tum has more pronounced anti-tumorigenic effect than applications of each factor alone, and that this strong anti-tumorigenic effect of ES + Tum is likely mediated by a combination of anti-angiogenic and direct anti-tumorigenic activities. Moreover, we show that ES + Tum therapy induces up-regulation of the prolactin receptor in GBM *in vivo* and that the activation of PL/PRLR signaling stimulates proliferation. Additional studies are necessary to elucidate whether the PRL/PRLR signalling pathway represents a novel target for therapeutic strategies aimed at developing effective treatments for GBM.

## Material and methods

### Expression vectors and transfection procedure

CMV (human cytomegalovirus) promoter driven plamids were used to generate expression vectors for angiogenic inhibitors. Murine ES was introduced into pcDNA3.1 plasmid (Invitrogen Life Technologies, Carlsbad, CA) as described previously [32]. The cDNA coding for Tum was obtained by RT-PCR from total RNA extracted from HDMECs (human dermal microvascular endothelial cells) using following primer-pair: forward-primer 5′ccgagctcggatccaggtttgaaaggaaaa3′ and reverse-primer 5′cgctcgagggtgtcttttcatgcacacct3′, and was cloned into pSecTag2/Hygro (Invitrogen Life Technologies). The cDNA encoding full length PRLR together with a C-terminal HA-tag was cloned in pcDNA3.1(−)/Hygro plasmid (Invitrogen Life Technologies). The PRLR cDNA was obtained by RT-PCR from total RNA extracted from the cell line MCF-7 using following primer-pair: forward-primer 5′aacactcgagaaggcagccaacatgaaggaaaat3′ and reverse-primer: 5′tgggtaccttaagcgtaatctggaacatcgtatgggtagtgaaaggagtgtgt3′. Porcine aortic endothelial (PAE) cells were transfected with 2 μg plasmid DNA (ES, Tum), and the glioma cell line G55 with 1 μg plasmid DNA (PRLR) using Lipofectamine Plus® (Invitrogen Life Technologies) according to manufacturer instructions. Positive cells were selected by application of the appropriate antibiotics, and again expanded.

### Cell culture and microencapsulation

Commercially available HUVECs and HDMECs were cultured in EGM-2 medium (Lonza, Basel, Switzerland) containing 2% fetal calf serum (FCS). PAE cells were maintained in F-12/HAM medium supplemented with 10% FCS. The human glioblastoma cell line G55 [50] was kindly provided by Prof. Katrin Lamszus from the Department of Neurosurgery, University Hospital Hamburg-Eppendorf, and cultured in Modified Eagle’s Medium supplemented with 10% FCS. All cells were maintained in 5% CO_2_/95% air atmosphere in a humidified incubator at 37°C. Wild-type or stably transfected PAE cells were encapsulated in Alginat microbeads as described previously [22,23]. Cells were resuspended in a 2% sodium alginate–saline solution (Pronova Ultra-Pure MVG; FMC BioPolymer AS d/b/a NovaMatrix, Sandvika, Norway) to a final concentration of 2 × 10^6^ cells/ml. For *in vitro* experiments conditioned medium (CM) was collected after a culture period of 48 hours. For long stimulation experiments CM was collected after a culture period of 4 days and subsequently diluted 1:3 with serum-reduced medium.

### Cell viability and proliferation assay

HUVEC and G55 cells (5 × 10^4^ per well) were seeded on 48-well tissue culture plates and incubated in basal medium or in CM or mixtures of CM from PAE-WT, PAE-Tum and PAE-ES cells additionally containing 4% FCS. Each stimulation experiment was performed in triplicate. After 24 and 48 hours of incubation at 37°C, cells were trypsinized and counted using the Vi-Cell XR (Beckman Coulter, Germany).

Cellular viability and proliferation was assessed using the WST-1 assay (Roche Applied Science, Mannheim, Germany) following the manufacturer´s instructions. Stably PRLR-transfected or mock-transfected G55-WT or G55 cells (8 × 10^3^ cells per well) were cultured under serum deprivation in presence of AG490 (0.1, 1 and 10 μM) and/or 2 nM prolactin (R&D Systems, Minneapolis, MN). To quantify cell viability, cells were incubated with WST-1 reagent for 1 h and the absorbance was measured using a plate reader at 450 nm (reference 650 nm). Each stimulation experiment was performed in quintuplicate. Cell viability of experimental cells was related to cell viability of control (untreated) cells, which was set to 100%.

### Apoptosis assay

G55 cells were seeded at subconfluent density into multiwell tissue culture plates. After culture overnight, cells were washed twice with PBS, and medium was replaced with CM from PAE-WT, PAE-Tum or PAE-ES cells, or a mixture of CM from PAE-Tum and PAE-ES cells. Incubations of cultures were continued for 24 hours before cells were analysed for apoptosis. For analysis, adherent cells were detached and pooled with floating cells. Apoptosis was assessed by flow cytometric analysis of cells stained with FITC-conjugated annexin-V and PI (BD Biosciences Pharmigen, San Diego, CA). Values represent the mean of three independent experiments.

### Western blotting

Supernatants of transfected PAE cells were tested for transgene expression using Western blot analysis. CM from PAE-ES cells was incubated overnight at 4°C with heparin agarose (Sigma-Aldrich, St. Louis, MO) for protein concentration. Supernatant of PAE-Tum cells were concentrated overnight at 4°C by Nickel Cam™ HC resin (Sigma-Aldrich) according to manufacturer instructions. ES, Tum and PRLR were detected by murine ES polyclonal antibody (R&D Systems) [24], His-probe polyclonal antibody (Santa Cruz Biothechnology, Santa Cruz, CA) [24] and HA antibody (H6908; Sigma-Aldrich), respectively. The signal was visualized by Lumigen™ PS-3 detection reagent (GE Healthcare, Buckinghamshire, UK)) and exposed to an Amersham Hyperfilm™ECL (GE Healthcare).

### In vitro wound healing assay

HDMEC cells (7× 10^5^) were cultured in 24-well tissue culture plates in endothelial growth medium with supplements. After reaching confluence each well was scratched with a standardized pipette tip, resulting in an EC-free wound. Medium was replaced with CM of WT or transfected PAE cells additionally supplemented with 4% FCS. Photographs of each well were taken direct after scratching and after 20 hours incubation. The width of the gap was determined using AxioVision40 V4.8 software (Carl Zeiss Imaging Solutions, Jena, Germany) and values representing the closing wound were compared between experimental groups. Values represent the mean of three independent experiments.

### In vivo tumor model

Animal experiments were conducted according to the UKCCR guidelines for the welfare of animals in experimental neoplasia [51]. G55 cells (1× 106) were subcutaneously injected into SCID mice. Microbeads containing 1 × 106 WT or transfected PAE cells were implanted at the same site 7 days later. In the combination groups 1 × 106 PAE cells producing each inhibitor were injected. Each experimental group consisted of 5 animals. After 10 days, animals were sacrificed and tumors were excised and weighed. One half of each tumor was fixed in 10% formaldehyde and embedded in paraffin for immunohistochemistry. The other half was frozen in liquid nitrogen and used for RNA isolation.

### Immunohistochemistry and immunofluorescence

Paraffin-embedded tissue samples were serially sectioned at a thickness of 5 μm, and every 20th section was used for analysis. Tissue sections were consecutively stained with hematoxylin and eosin. Blood vessels were visualized using murine polyclonal CD31 antibody [52] (Dianova, Hamburg, Germany). Monoclonal Ki67 [52], polyclonal prolactin receptor and M30 CytoDEATH antibodies were purchased from Dako (Dako Denmarck A/S, Glostrup, Denmark), abcam (Cambridge, UK) and Roche Applied Science respectively. Immunhistochemical staining was performed as previously described [52]. For double immunhistochemical analyses, M30 and PRLR antibodies were visualized with Diaminobenzidine (DAB) and 3-Amino-9-ethylcarbazole (AEC), respectively. A blocking step in between using the Avidin-Biotin Blocking Kit (Vector Laboratories, Inc., Burlingame, CA) was performed. For immunofluorescence detection of PRLR in G55 cells, 3× 10^5^ cells were seeded on chamber slides and treated with CM or mixtures of CM from PAE-WT, PAE-Tum and PAE-ES cells for 3 days. After fixation with cold ice methanol, staining was performed as previously described [52]. Microvessel density was quantified by counting CD31-positive vessels in 10 arbitrarily chosen visual fields (10 × magnification) per tumor from totally 4 to 5 tumors from each experimental animal group using AxioVision40 V4.8 software (Carl Zeiss Imaging Solutions, Jena, Germany).

### TUNEL assay of apoptotic cells

For the *in situ* detection of fragmented DNA, tissue sections were subjected to terminal deoxynucleotidyl transferase dUTP nick end labeling (TUNEL) using the *in situ cell death detection kit, POD* (Roche Diagnostic GmbH) according to the manufacturer´s instructions. Nuclei were counterstained with haematoxylin. TUNEL-negative nuclei were stained blue, while TUNEL-positive nuclei were stained brown.

### RNA isolation and microarray analysis

Frozen tumor samples were homogenized with a micro tissue disintegrator. Tissue homogenates were first treated with Tri®Reagent (Sigma-Aldrich) for RNA Isolation succeeded by purification with the RNeasy Mini Kit (Qiagen, Hilden, Germany) following manufactures protocols. Quality and concentration of isolated RNA was determined using the Agilent RNA 6000 Nano Kit (Agilent Technologies Inc., Waldbronn, Germany) and NanoDrop6000 Photometer (Peqlab Biothechnologie GmbH, Erlangen, Germany). From each experimental animal group, three RNA samples were selected for further microarray analyses. Sense strand cDNA was generated from 100 ng total RNA using the Ambion WT Expression Kit (Ambion Inc., Applied Biosystems, Austin, TX). Procedures for labelling, fragmentation and hybridization were performed using the Terminal Labeling Kit and Hybridization, Wash and Stain Kit following Affymetrix protocols. All experiments were performed using Affymetrix Human Gene 1.0 ST Array containing 28.869 genes (Affymetrix Inc., Santa Clara, CA). Microarrays were scanned with the GeneChip Scanner 3000 7G (Affymetrix Inc.) using the GeneChip Command Console 3.0 (Affymetrix Inc.). The signals were processed using Genelevel RMA Sketch algorithm with following software: Affymetrix® Expression Console™ 1.1 software. Comparison analyses were carried out with a T-Test (between subjects) (TIGR, The Institute for Genomic Research, MeV 4).

### Statistical analysis

Statistical analyses were performed using the Statistical Package for Social Sciences (SPSS) program (SPSS Inc. Chicago, IL, USA), version 15 with a Mann–Whitney-U-Test for tumor growth, microvessel density data and wound assays and with the unpaired Student *t* test with Welch correction for proliferation experiments. Probability value (p-value) ≤ 0.05 was considered statistically significant.

## Abbreviations

CGT: Cilengitide; CM: Conditionated medium; EC: Endothelial cells; ES: Endostatin; GBM: Glioblastoma Multiforme; HDMEC: Human dermal microvascular endothelial cells; PAE: Porcine aortic endothelial cells; PRL: Prolactin; PRLR: Prolactin receptor; Tum: Tumstatin.

## Competing interests

The authors declare that they have no competing interests.

## Authors’ contributions

LOF, JW, GS and WF designed this research work. LOF, JH and MK performed experiments and analysed data in this manuscript. LOF, JW, UB, EMMP, WF, CB and GS interpreted the data and wrote the manuscript. All authors read and approved the final manuscript.

## Supplementary Material

Additional file 1: Figure S1 Expression analyses of αVβ3 and α5β1 integrins in G55 cells. Endogenous expression of αVβ3 and α5β1 integrins in G55 cells was detected (PDF 50 kb) at the mRNA level using RT-PCR (A) and at the protein level using Flow Cytometry Analyses (B). Control Integrin αVβ3 Control Integrin α5β1. Click here for file

Additional file 2: Table S1 Differentially regulated genes in G55 tumos after treatment with angiogenic inhibitors. Click here for file

Additional file 3: Figure S2 Effect of PRL on glioma cell proliferation. Endogeneous PRLR expression at mRNA level was detected in both G28 and G55 cells using RT-PCR (A). Human breast cancer cell line T47D was used as a positive control. Prolactin (PRL) stimulated cell proliferation of both cell lines in a dose-dependent manner (B). Bars represent the mean values ± SE (n = 5-6); * on bars indicates significant differences vs. control (p < 0. 05). Click here for file
