# Supplementary material for: Combination therapy targeting integrins reduces glioblastoma tumor growth through antiangiogenic and direct antitumor activity and leads to activation of the pro-proliferative prolactin pathway
Source: Mol Cancer. 2013 Nov 20;12:144. doi: 10.1186/1476-4598-12-144 (PMC4176123; doi:10.1186/1476-4598-12-144)
Supplement: Additional file 1: Figure S1 — Expression analyses of αVβ3 and α5β1 integrins in G55 cells. Endogenous expression of αVβ3 and α5β1 integrins in G55 cells was detected (PDF 50 kb) at the mRNA level using RT-PCR (A) and at the protein level using Flow Cytometry Analyses (B). Control Integrin αVβ3 Control Integrin α5β1. [file 1476-4598-12-144-S1.pdf]

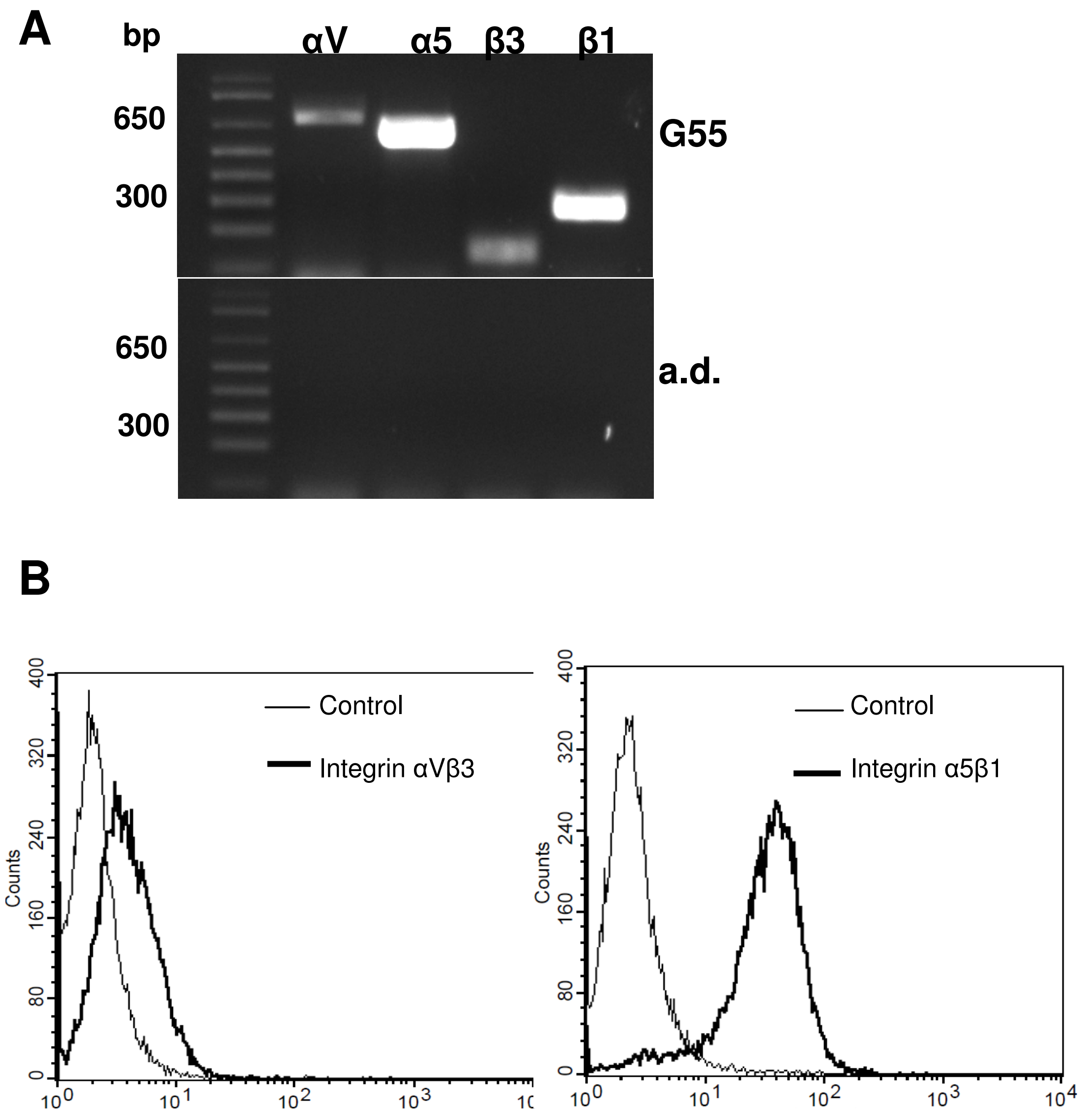

Supplementary Figure 1S. **Expression analyses of αvß3 and α5ß1 integrins in G55 cells.** Endogenous expression of αvß3 and α5ß1 integrins in G55 cells was detected at the mRNA level using RT-PCR (A) and at the protein level using Flow Cytometry Analyses (B).
