# Supplementary material for: Combination therapy targeting integrins reduces glioblastoma tumor growth through antiangiogenic and direct antitumor activity and leads to activation of the pro-proliferative prolactin pathway
Source: Mol Cancer. 2013 Nov 20;12:144. doi: 10.1186/1476-4598-12-144 (PMC4176123; doi:10.1186/1476-4598-12-144)
Supplement: Additional file 2: Table S1 — Differentially regulated genes in G55 tumos after treatment with angiogenic inhibitors. [file 1476-4598-12-144-S2.pdf]

Table 1S Differentially regulated genes in G55 tumos after treatment with angiogenic inhibitors

Gene	Name	Affimetrix ID	SLR
Tumstatin group vs. WT group			
MXD1	MAX dimerization protein 1	8042503	+0,3
PRL	Prolactin	8124185	+0,3
PPARA	Peroxisome proliferator-activated receptor alpha	8073826	+0,3
Tumstatin + Endostatin group vs. WT group			
PRLR	Prolactin receptor	8111490	+0,3
FRMPD1	FERM and PDZ domain containing	8155284	-0,3
IL1F9	Interleukin 1 family, member 9	8044541	-0,2
