# Supplementary material for: Combination therapy targeting integrins reduces glioblastoma tumor growth through antiangiogenic and direct antitumor activity and leads to activation of the pro-proliferative prolactin pathway
Source: Mol Cancer. 2013 Nov 20;12:144. doi: 10.1186/1476-4598-12-144 (PMC4176123; doi:10.1186/1476-4598-12-144)
Supplement: Additional file 3: Figure S2 — Effect of PRL on glioma cell proliferation. Endogeneous PRLR expression at mRNA level was detected in both G28 and G55 cells using RT-PCR (A). Human breast cancer cell line T47D was used as a positive control. Prolactin (PRL) stimulated cell proliferation of both cell lines in a dose-dependent manner (B). Bars represent the mean values ± SE (n = 5-6); * on bars indicates significant differences vs. control (p < 0. 05). [file 1476-4598-12-144-S3.pdf]

A

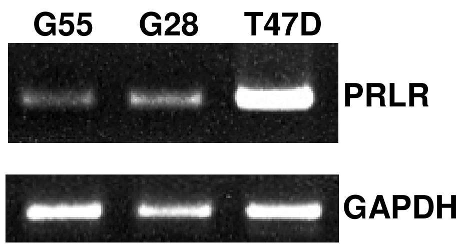

B

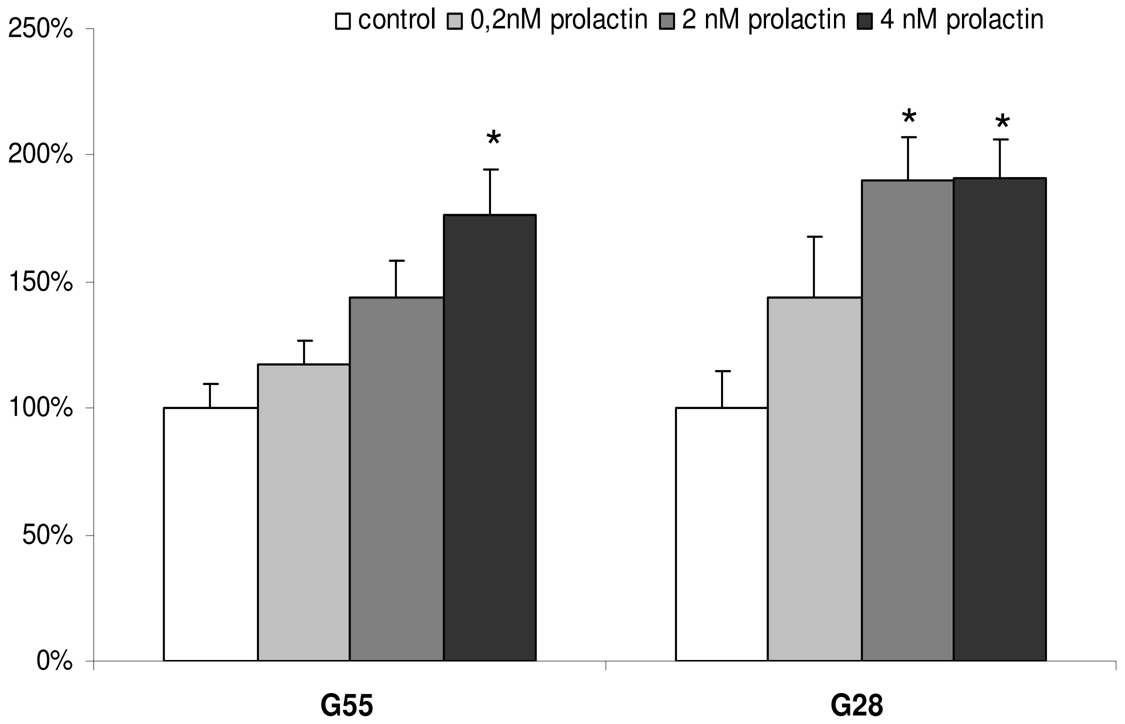

Supplementary Figure 2S. **Effect of PRL on glioma cell proliferation**. Endogeneous PRLR expression at mRNA level was detected in both G28 and G55 cells using RT-PCR (A). Human breast cancer cell line T47D was used as a positive control. Prolactin (PRL) stimulated cell proliferation of both cell lines in a dose-dependent manner (B). Bars represent the mean values ± SE (n=5-6); * on bars indicates significant differences vs. control (p<0. 05) .
